# Spatial and temporal localization of cell wall associated pili in *Enterococcus faecalis*

**DOI:** 10.1111/mmi.15008

**Published:** 2022-12-07

**Authors:** Pei Yi Choo, Charles Y. Wang, Michael S. VanNieuwenhze, Kimberly A. Kline

**Affiliations:** ^1^ Singapore Centre for Environmental Life Sciences Engineering Nanyang Technological University Singapore Singapore; ^2^ School of Biological Sciences Nanyang Technological University Singapore Singapore; ^3^ Department of Chemistry Indiana University Bloomington Indiana USA; ^4^ Department of Microbiology and Molecular Medicine University of Geneva Geneva Switzerland

**Keywords:** *Enterococcus faecalis*, pili, sortase, spatiotemporal localization

## Abstract

*Enterococcus faecalis* virulence requires cell wall‐associated proteins, including the sortase‐assembled endocarditis and biofilm associated pilus (Ebp), important for biofilm formation in vitro and in vivo. The current paradigm for sortase‐assembled pilus biogenesis in Gram‐positive bacteria is that sortases attach substrates to lipid II peptidoglycan (PG) precursors, prior to their incorporation into the growing cell wall. Contrary to prevailing dogma, by following the distribution of Ebp and PG throughout the *E. faecalis* cell cycle, we found that cell surface Ebp do not co‐localize with newly synthesized PG. Instead, surface‐exposed Ebp are localized to the older cell hemisphere and excluded from sites of new PG synthesis at the septum. Moreover, Ebp deposition on the younger hemisphere of the *E. faecalis* diplococcus appear as foci adjacent to the nascent septum. We propose a new model whereby sortase substrate deposition can occur on older PG rather than at sites of new cell wall synthesis. Consistent with this model, we demonstrate that sequestering lipid II to block PG synthesis via ramoplanin, does not impact new Ebp deposition at the cell surface. These data support an alternative paradigm for sortase substrate deposition in *E. faecalis*, in which Ebp are anchored directly onto uncrosslinked cell wall, independent of new PG synthesis.

## Graphical abstract

*Enterococcus faecalis* endocarditis and biofilm associated pili (Ebp) are surface exposed virulence factors that are attached onto the cell wall by sortase enzymes. Here, we show that pili are surface exposed sequentially towards the cell pole at the cell hemispheres in a cell wall age‐dependent manner. In addition, we propose an alternative cell wall anchoring pathway where pili are directly anchored onto mature uncrosslinked cell wall, independent of new cell wall synthesis at the septum.

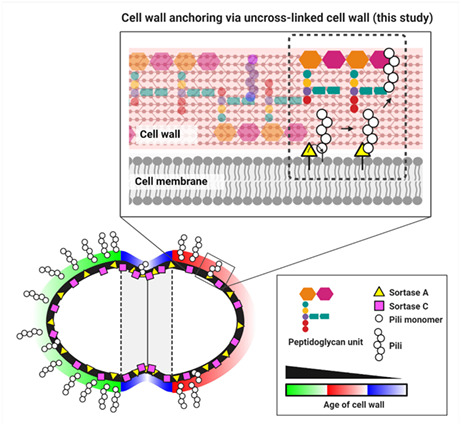

## 1 INTRODUCTION

*Enterococcus faecalis* is a Gram‐positive ovococcal bacteria commonly found in the gastrointestinal tracts of humans and other mammals. *E. faecalis* is a public health concern due to its resistance to many antimicrobial drugs (Arias & Murray, 2012). It is an opportunistic pathogen capable of causing life‐threatening infections in humans such as infective endocarditis, bacteremia, and urinary tract infection (UTI) (Hidron et al., 2008; Murdoch et al., 2009; Patterson et al., 1995; Weiner‐Lastinger et al., 2019). Its ability to colonize and infect the human host involves multiple virulence factors that are responsible for adhesion and biofilm formation, including aggregation substance (AS), collagen binding protein (Ace), enterococcal surface protein (Esp), and endocarditis and biofilm‐associated pilus (Ebp) (Hendrickx et al., 2009). Unlike Gram‐negative bacteria, which possess an outer membrane, Gram‐positive bacteria have a thick peptidoglycan (PG) cell wall that acts as a scaffold for the processing and attachment of these virulence factors. A hallmark of these cell‐wall associated virulence factors is their C‐terminal cell wall sorting signal, which consists of an LPXTG motif, a hydrophobic domain, and a positively charged cytoplasmic tail (Schneewind et al., 1992). The enzyme responsible for the recognition and anchoring of these surface proteins to the peptidoglycan is the housekeeping enzyme, sortase A (SrtA) (Mazmanian et al., 1999; Ton‐That et al., 1999).

Ebp are well‐characterized cell wall‐attached surface proteins in *E. faecalis*. Ebp are important for biofilm formation and are implicated in endocarditis and UTI (Nallapareddy et al., 2011; Sillanpaa et al., 2013). Each Ebp is composed of three pilin subunits (EbpA, EbpB, and EbpC), where EbpA and EbpB are minor subunits that form the pilus tip and base, respectively, while EbpC is the major subunit that makes up the pilus backbone (Nallapareddy et al., 2006; Nielsen et al., 2013). Ebp encoding genes are in an operon together with sortase C (*srtC*), which is the sortase responsible for Ebp subunit polymerization (Nallapareddy et al., 2011). Assembled pili are then anchored onto the cell wall by SrtA. Through this sorting mechanism, fully polymerized and cell wall‐anchored Ebp become surface‐exposed and can facilitate adhesion to abiotic and biotic surfaces.

SrtA is a membrane‐anchored transpeptidase conserved in most Gram‐positive bacteria (Marraffini et al., 2006). SrtA is focally enriched at the septum of *E. faecalis* cells (Kline et al., 2009). SrtA substrates are translocated across the cell membrane by the general Sec secretion machinery, whereupon they are transiently membrane‐associated with the cell membrane via their C‐terminal transmembrane domain. The LPXTG motif within the membrane‐associated substrates is recognized and cleaved by SrtA between the threonine and glycine residue. Cleavage results in a covalent linkage between the active site cysteine residue of SrtA and the carbonyl group of the threonine residue of the substrate to generate an acyl‐enzyme intermediate. This intermediate is then covalently captured by a lipid II cell wall precursor and is subsequently incorporated into the growing peptidoglycan layer via well‐established transglycosylation and transpeptidation reactions (Perry et al., 2002; Ruzin et al., 2002; Ton‐That & Schneewind, 1999). The mechanism of SrtA has largely been defined in *Staphylococcus aureus*, which has served as a model organism for Gram‐positive SrtA function. Although there is strong evidence that the crossbridge on lipid II is the anchoring site for sortase substrates, and the prevailing model is that the lipid II precursor is the SrtA target, the possibility of substrate anchorage onto the crossbridge of older uncrosslinked PG has not been eliminated.

The enterococcal cell wall, like all Gram‐positive bacteria, is composed mainly of PG, wall teichoic acid, and lipoteichoic acid (Rajagopal & Walker, 2017). The PG layers sit directly above the phospholipid bilayer, forming a lattice structure that protects the cell from osmotic stress and pressure. A single *E. faecalis* PG unit is made up of disaccharide N‐acetylmuramic acid‐N‐acetylglucosamine (NAM‐NAG) with a pentapeptide stem attached to NAM, and an L‐Ala‐L‐Ala crossbridge attached to the ε‐amino group of the stem lysine residue (Schleifer & Kandler, 1972). PG subunits are polymerized into glycan chains by the glycosyltransferase activity of penicillin binding proteins (PBPs) and by shape, elongation, division, and sporulation (SEDS)‐family proteins, RodA and FtsW (Meeske et al., 2016). Adjoining glycan chains are then crosslinked via transpeptidation, where the transpeptidase activity of PBPs forms a peptide bond between the L‐alanine crossbridge and the D‐alanine residue at the fourth position of the pentapeptide stem. During crosslinking, the terminal D‐alanine of the pentapeptide stem is removed. Although the general PG subunit is similar for most Gram‐positive bacteria, the amino acid sequence of the crossbridge can vary. The crossbridge of *E. faecalis* consists of two L‐alanines while the crossbridges of *Enterococcus faecium*, *Streptococcus pneumoniae*, or *S. aureus* are composed of a single D‐aspartate, L‐alanine‐L‐alanine or L‐serine‐L‐alanine, or five glycines, respectively (Bellais et al., 2006; Fiser et al., 2003; Schneider et al., 2004). The differences in crossbridge length in turn affect the extent of cell wall crosslinking, whereby a longer crossbridge is associated with a more crosslinked cell wall and vice versa. For example, the percentage of crosslinking of *S. aureus* ranges from 74%–92% (Vollmer & Seligman, 2010), whereas the percentage crosslinking in *E. faecalis* and *S. pneumoniae* is approximately 48% and 35%, respectively (Bui et al., 2012; Yang et al., 2017). Therefore, whether SrtA functions differently with these varying PG crossbridge compositions remains an open question.

While pilus assembly, sorting machinery, and cell wall synthesis are interlinked and well‐studied as individual processes, little is known about the spatiotemporal interplay between these mechanisms. Moreover, despite differences in cell wall structure among Gram positive bacteria, the main paradigm for cell wall protein surface deposition is based on studies carried out in *S. aureus*. Here, we sought to bridge the gap between cell wall protein anchoring and cell wall synthesis by investigating the spatial and temporal distribution of surface‐exposed pili in *E. faecalis*. Since *E. faecalis* sortases are focally enriched at the cell septum (Kline et al., 2009), which coincides with the site of new cell wall synthesis, we hypothesized that new Ebp would also emerge focally at the division septum. To our surprise, we found instead that Ebp emerge and localize at the cell periphery, predominantly saturating one hemisphere of the diplococcus, and are excluded from the cell septum. Our data supports that *E. faecalis* SrtA substrates become incorporated at sites of uncrosslinked older PG at the cell periphery, and not at sites of new wall synthesis, expanding the current paradigm for SrtA activity in non‐model bacteria.

## 2 RESULTS

### 2.1 Cell surface‐exposed Ebp are septum excluded and temporally deposited towards the pole

**FIGURE 1:**
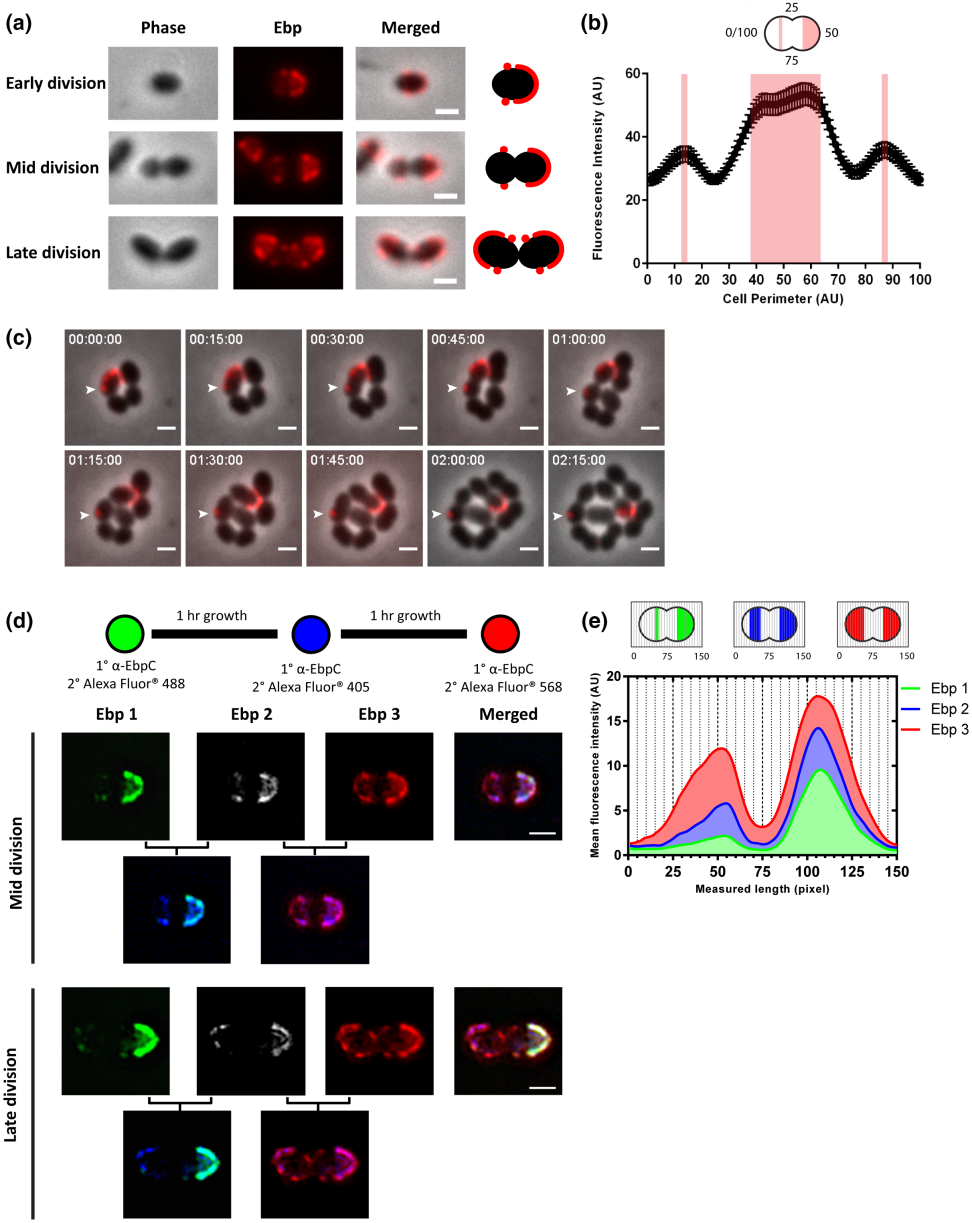
Distribution of surface‐exposed Ebp in *Enterococcus faecalis*. (a) Representative immunofluorescence (IF) labelling of surface‐exposed Ebp in *E. faecalis* at early, mid and late division. Scale bar, 1 μm. (b) Quantification of Ebp fluorescence intensity along the cell periphery (0–100) of mid division cells starting from the cell pole where 25 and 75 correspond to the cell septum (*n* = 92). The shaded areas on the graph correspond to one hemisphere and two foci adjacent to the equatorial ring with peak fluorescence intensity. (c) Time‐lapse of live *E. faecalis* cells pre‐labeled for Ebp. Scale bar, 1 μm. (d) Chase labelling of Ebp via immunofluorescence at 1 h growth intervals using green, blue and red fluorescent conjugated secondary antibodies. Images on the second row are merged images of either Ebp 1 and Ebp 2 or Ebp 2 and Ebp 3. Representative mid and late division phase cells are shown. Scale bar, 1 μm. (e) Quantification of SIM images where individual cells were cropped and orientated along the longitudinal cell axis such that the new cell hemisphere is always on the left. Ebp fluorescence intensity values for each diplococcus were averaged in each the vertical plane of the cell (depicted by vertical dotted lines) and plotted against the length of the cell. The average compiled fluorescence for 71 cells over three independent experiments are shown.

Sorting and cell surface‐exposure of Enterococcal pili facilitate virulence during infection. We visualized the distribution of fluorescently labeled *E. faecalis* pili at three different growth phases, defined as (1) early division elongated monococci which have not yet undergone septation (1 μm–1.5 μm in length), (2) mid division diplococci which are undergoing elongation and septum constriction (1.5 μm–2 μm), and (3) late division cells consisting of two daughter cells just before separation (>2 μm). During each growth phase, we observed EbpC labelling only at the cell hemispheres and at sites adjacent to the equatorial ring, but not at the septum, a pattern we refer to as “septum excluded” (Figure 1a). Furthermore, we observed that one hemisphere of the cell is always more saturated with Ebp than the other. The hemispherical saturation and septum excluded localization patterns were seen in early and mid‐division phase cells. However, in late division cells, we observed EbpC fully covering both hemispheres with additional foci adjacent to the nascent septum, mirroring each other (Figure 1a). The transition from asymmetric distribution of Ebp in the mid‐division phase to the symmetric Ebp distribution in late division phase between the two hemispheres hinted that the foci adjacent to the septum may eventually seed the coverage of the entire hemisphere. To quantify the localization pattern of EbpC, we plotted the Ebp fluorescence intensity against the cell perimeter in mid‐division phase cells and observed that the fluorescence intensity is lowest at positions that correspond to the septum (Figure 1b). Low septal fluorescence intensity is consistent with our observation that the localization of cell wall associated Ebp is septum excluded. Moreover, the fluorescence intensity is highest from positions 38 to 63, which corresponds to one hemisphere of the cell, again consistent with our observation that one side of the cell is more saturated with Ebp than the other. Two smaller peaks were observed at positions 13 and 87, which correspond to the foci located adjacent to the septum on the other hemisphere of the cell (Figure 1b). Because the maximum fluorescence intensity of these two foci is lower than the other hemisphere, we hypothesized that these foci represent newly emerged Ebp and that additional Ebp would continue to emerge and eventually complete the hemispherical coverage. Overall, these findings indicate that surface‐exposed pilus localization is septum excluded and that Ebp decoration predominates at one hemisphere of the cell.

Because we observed that Ebp become surface exposed and saturate one cell hemisphere before the other, we proposed two possible scenarios to describe how one hemisphere becomes fully piliated. In the first scenario, the foci adjacent to the septum remain immobile and newer pili emerge in front of them, proceeding towards the pole, where they eventually converge to cover the hemisphere. In the second scenario, the foci migrate towards the pole while new Ebp continue to emerge behind the migrating foci from the site adjacent to the septum. To determine which of these two hypotheses could explain temporal pilus deposition on the cell wall, we performed time‐lapse fluorescence microscopy on live EbpC‐labeled cells. EbpC labelling was performed prior to mounting the cells onto a BHI agarose gel pad. We tracked the original fluorescent Ebp foci on the cell and observed that as the cell elongates and divides, the single focus does not move along the cell periphery. Instead, the stained EbpC focus remains where it was originally labeled, while new cell material is synthesized at the septum (Figure 1c). This lack of focus movement suggested that once deposited on the cell wall, Ebp are immobile and newer Ebp are likely deposited towards the cell pole to achieve their hemispherical localization pattern, supporting our first proposed scenario. To track newly emerged Ebp that are predicted to emerge towards the cell pole, we performed an Ebp pulse chase labelling experiment where Ebp were labeled three consecutive times via immunofluorescence using green, blue, and red fluorescently labeled secondary antibodies sequentially with a washout step and 1 h of growth between each labelling. We imaged the cells using structured illumination microscopy (SIM) and observed that Ebp were labeled in a sequential manner towards the pole (Figure 1d). In the hemisphere where the two Ebp foci were initially labeled in green, we observed blue‐labeled Ebp (Ebp 2) overlapping with the older green‐labeled Ebp (Ebp 1), in addition to new blue‐label localizing to segments adjacent to the green foci. On the same hemisphere, red‐labeled Ebp (Ebp 3), representing the newest Ebp deposited on the cell surface, overlapped with both green and blue and covered the entire cell hemisphere. We quantified the mean fluorescence intensity of all three channels within each vertical axis, along the cell length of mid‐division phase cells (Figure 1e). Individual cells were oriented such that the left cell hemisphere is less saturated than the right. Consistent with visual inspection, we observed accumulation of fluorescence intensities from Ebp 1 to Ebp 2 and from Ebp 2 to Ebp 3 at the left cell pole, indicating that new pili were emerging during each labelling period. The build‐up of fluorescent signal from sections 25 to 50 on the new hemisphere with each progressive labelling suggests that newer pili are appearing in a non‐random manner, with increasing Ebp fluorescence towards the pole over time. Furthermore, the overall fluorescence intensity of surface‐exposed Ebp also increased at each time point, suggesting that new Ebp is not only emerging towards the pole but also saturating the older Ebp deposition sites. To test if other cell wall anchored substrates were exposed in a similar manner, we performed a similar chase labelling experiment on aggregation substance (AS) and observed similar sequential deposition of AS on the cell (Figure S1). These data were consistent with our observation on live cells that Ebp deposited on the cell wall are fixed in space and that newer Ebp are deposited in a temporal manner towards the cell pole where, over time, newer Ebp are exposed closer to the pole while older Ebp remain closer to the equatorial rings until eventually, the whole hemisphere is saturated with Ebp. These data also hinted that Ebp deposition and cell wall synthesis are coordinated where new Ebp may appear together with newly synthesized cell wall.

### 2.2 New cell wall is synthesized at the septum and is driven towards the cell hemisphere as the cell elongates

**FIGURE 2:**
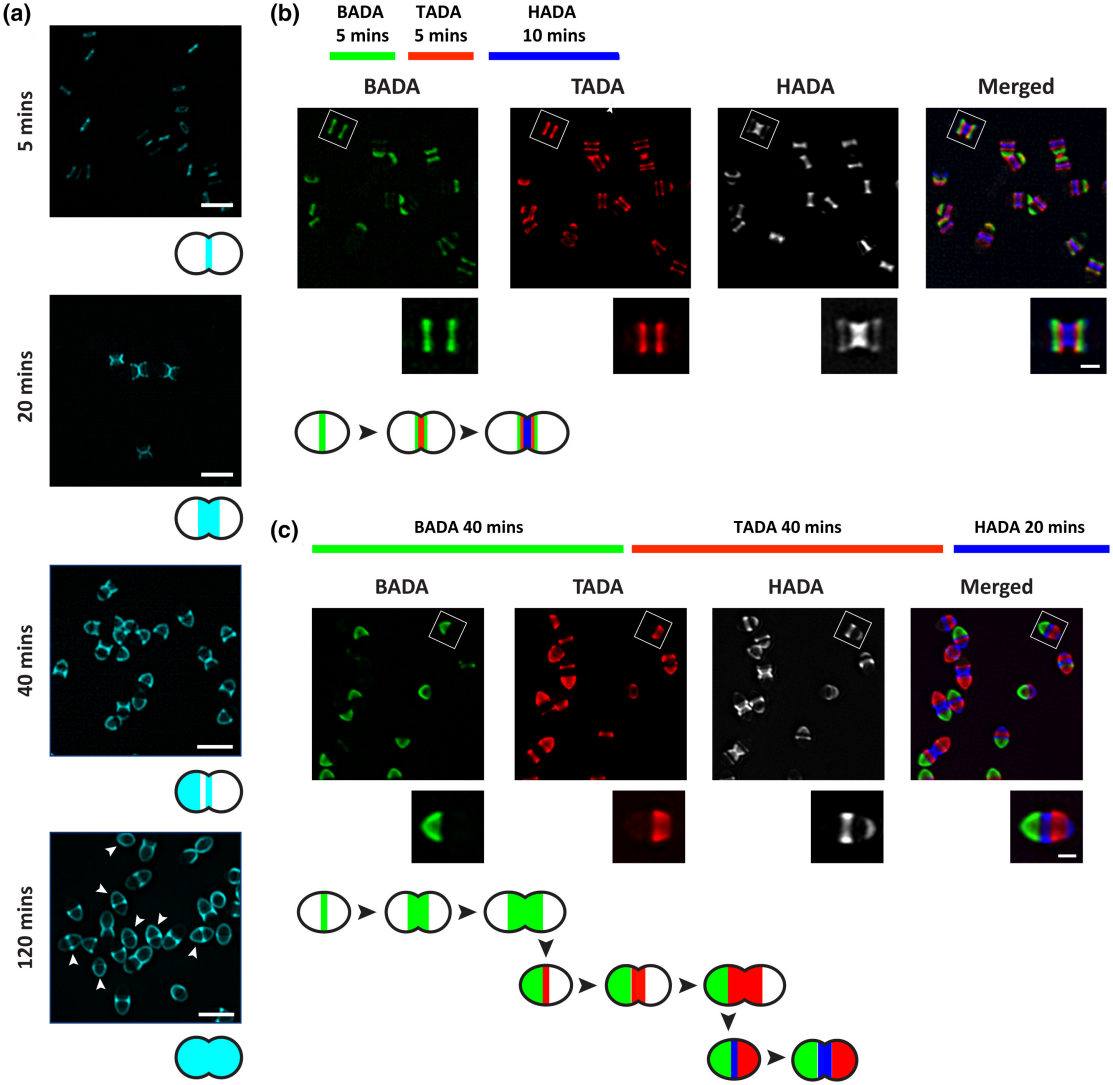
Fluorescent D‐amino acid (FDAA) labeling of *Enterococcus faecalis* cell wall throughout the cell cycle. (a) Representative images of HCC‐amino‐D‐alanine (HADA) labeled *E. faecalis* cell wall, pseudocolored cyan, at various time points. Cells were grown to exponential phase and labeled with HADA for 5, 20, 40 and 120 min. White arrows indicate cells that are fully labeled with HADA. Scale bar: 1 μm. (b and c) representative images after sequential labelling of *E. faecalis* cell wall at short (b) and long (c) pulses and imaged by SIM. In (b), cells were labeled with BADA (5 min), TADA (5 min) and HADA (10 min). In (c), cells were labeled with BADA (40 min), TADA (40 min) and HADA (20 min). Scale bar: 0.5 μm. Schematics of representative cells are shown at the bottom of each panel.

The current paradigm in Gram‐positive bacteria, largely defined in *S. aureus*, is that SrtA substrates are anchored to the cell wall via lipid II, a cell wall precursor found at the septum (Ton‐That et al., 1997; Ton‐That & Schneewind, 1999). Similarly, we postulated that, in *E. faecalis*, sortase‐anchored Ebp are attached to lipid II and become surface exposed as this lipid II‐substrate intermediate is incorporated into the growing PG cell wall. To test this, we first characterized *E. faecalis* cell wall synthesis dynamics using fluorescent D‐amino acid (FDAA) probes. FDAAs are fluorescently‐modified D‐amino acids that are actively incorporated into the cell wall by D,D‐ and L,D‐transpeptidases (Hsu et al., 2017; Kuru et al., 2015). We incubated mid log phase cells with HADA, a blue‐emitting FDAA, for 5, 20, 40, or 120 min and visualized the staining patterns using SIM (Figure 2a). After 5 min of HADA exposure, we observed cell wall labelling exclusively at the septum, indicating that new cell wall is synthesized at mid cell, as expected and reported for other related species (Boersma et al., 2015; Hsu et al., 2019). After 20 min of HADA exposure, we observed a distinctive “cross‐like” localization pattern where cell wall was stained from the equatorial ring to the septum and unlabelled at both hemispheres (Figure 2a). The cross‐like localization pattern supports our initial observation that new cell wall is synthesized at the mid‐cell. By contrast, after 40 min of labelling, most cells were stained at the septum and one side of the hemisphere while the other hemisphere remained unlabelled (Figure 2a). This differential distribution of PG staining is consistent with one half of each diplococcus being more mature than the other. After 120 min of HADA exposure, most cells were uniformly labeled, indicating that they have gone through at least 2 replication cycles (Figure 2a). The cell wall labelling patterns observed after HADA labelling for different durations led us to conclude that PG incorporation in *E. faecalis* is coordinated asymmetrically such that new PG is always synthesized at midcell and older PG is consistently, constantly, and evenly pushed out by newer cell wall with little or no intercalation of new and old cell wall material.

To spatially distinguish between old and new cell wall, and to determine the temporal distribution of PG, we next performed sequential labelling of new PG using three different colored FDAAs. A series of short pulses was carried out, in which we first incubated the cells for 5 min with a green FDAA, BODIPY‐FL 3‐amino‐d‐alanine (BADA) followed by 5 min with a red FDAA, TAMRA 3‐amino‐D‐alanine (TADA) and lastly, 10 min with HADA (Figure 2b). The three stains appeared as distinct fluorescent bands parallel to each other on the cell with minimal overlap, suggesting immobility of the cell wall PG after it is incorporated. BADA and TADA fluorescent bands appeared narrower than that of HADA's, suggesting splitting of these fluorescent bands at midcell as newer PG is synthesized and incorporated at the septum. Furthermore, BADA labeled PG formed the outermost bands, flanking both TADA and HADA labeled PG with TADA sandwiched between BADA and HADA (Figure 2b). This chronological labelling pattern supports our hypothesis that PG at midcell is pushed away from the septum as the cell elongates and divides. We further demonstrated the ability to visualize the older hemisphere of the cell by extending the serial FDAA labelling of BADA, TADA and HADA to 40 min, 40 min, and 20 min, respectively (Figure 2c). We chose 40 min for the first two pulses because we observed single hemispherical PG staining for this staining duration and if our hypothesis that one hemisphere of a diplococcus is always older than the other is true, we predicted that the older hemisphere of the cell will be labeled green, the other hemisphere labeled red, and the middle of the cell labeled blue. Consistent with this prediction, BADA labelling was seen predominantly at one hemisphere while TADA labeled the other cell hemisphere, and HADA staining was observed in the middle of the cell (Figure 2c). These data demonstrate that new cell wall synthesis in *E. faecalis* occurs strictly at the septum, where the older PG will be pushed towards the pole as the cell elongates and divides. Furthermore, there is minimal overlap between the old and new cell wall, and the older hemisphere is readily identified.

### 2.3 Surface‐exposed Ebp does not co‐localize with newly synthesized cell wall

**FIGURE 3:**
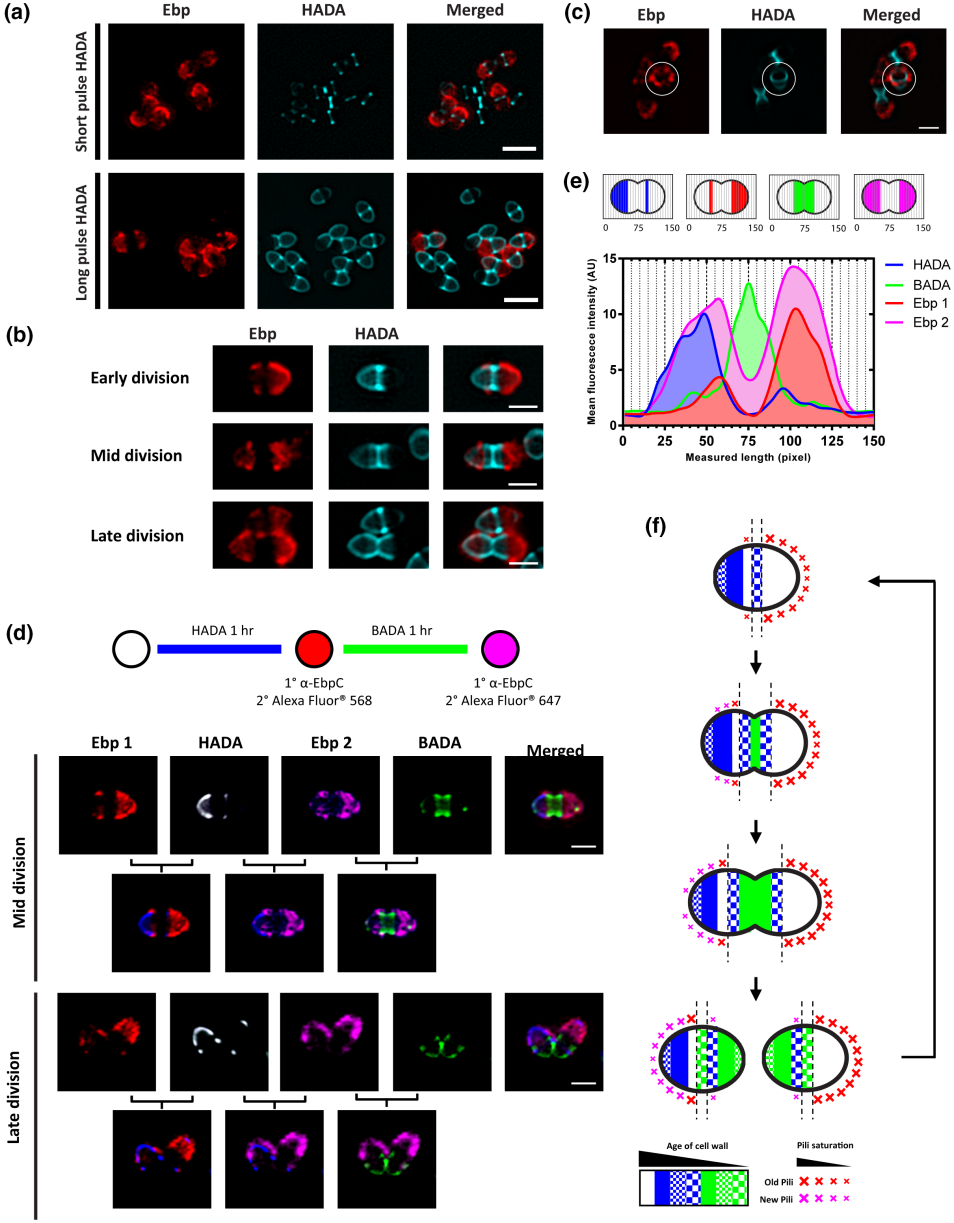
Co‐staining of peptidoglycan and Ebp in *Enterococcus faecalis*. (a and b) exponential growing cells were labeled with HADA for short (5 min) or long (120 min) pulse, stained for Ebp and imaged via SIM. Scale bar: 2 μm. (b), representative image of cells at early, mid and late division phases are shown. Scale bar:1 μm. (c) Representative image of cell tilted at an angle (circled), revealing multiple circumferential Ebp foci. Scale bar: 1 μm. (d) Representative image of mid and late division cells after undergoing HADA‐Ebp 1‐BADA‐Ebp 2 chase labelling. Cells were labeled with HADA for 1 h and then immunolabeled for Ebp using a red secondary antibody, followed by 1 h growth in BHI with BADA and then labeled for Ebp again using a far‐red secondary antibody. Scale bar: 1 μm. (e) Fluorescence intensity plot of HADA (old cell wall), old (Ebp 1), BADA (new cell wall) and new Ebp (Ebp 2) plotted against the cell length (*n* = 61) over 3 independent experiments. (f) Proposed model of pili exposure in relation to cell wall synthesis during cell division. Cells are segmented by age of the cell wall as determined in Figure 2, where white is the oldest followed by blue (solid), blue (small checker board), blue (large checker board), green (solid), green (small checker board) and green (large checker board). Sites of old and newly exposed pili are labeled red and magenta respectively. Initially, pili are saturated at the older hemisphere of the cell and appear as two foci adjacent to the septum on the younger hemisphere. As the cell elongates, new pili on the younger hemisphere are exposed sequentially towards the cell pole in a cell wall age dependent manner. The younger cell hemisphere is eventually saturated with surface‐exposed pili as the cell wall matures.

Ebp is a surface‐exposed virulence factor that is covalently attached onto the cell wall by SrtA (Nielsen et al., 2013). Based on studies in *S. aureus* (Perry et al., 2002; Ton‐That et al., 1997; Ton‐That & Schneewind, 1999), we postulated that Ebp is similarly attached to lipid II cell wall precursors at the septum prior to surface exposure. To test if Ebp exposure coincides with the new cell wall, we performed co‐localization studies by co‐staining both PG and pili (Figure 3a,b). We performed both short and long incubations with HADA to determine if there were any differences in co‐localization patterns. Surprisingly, surface exposed Ebp did not co‐localize with the newly synthesized labeled cell wall regardless of the length of HADA exposure. Instead, we observed Ebp staining where HADA labelling was absent. Ebp labelling also appeared more saturated at the older hemisphere. In addition, cell wall and Ebp co‐staining, when viewed at an angle, revealed that the two foci observed in cross sections exist as multiple circumferential foci (Figure 3c), suggesting that there are multiple Ebp anchoring points at the cross section of the cell. Cell wall and Ebp co‐staining results illustrate that a mature cell wall may be required before *E. faecalis* pilus become surface exposed and accessible to antibody labelling.

To test if PG synthesis precedes Ebp surface exposure, we performed a HADA‐Ebp‐BADA‐Ebp chase experiment. The experimental design was similar to the above assay, but included additional steps where cells were allowed to grow in BHI for an hour in the presence of BADA to label new cell wall and then stained again for Ebp with a far‐red fluorescent secondary antibody. When cells were exposed to HADA for 1 h, co‐stained with Ebp (Ebp 1), allowed to grow, and then stained a second time for Ebp (Ebp 2), we saw that newly labeled Ebp 2 overlapped with the older HADA‐labeled cell wall (Figure 3d). We quantified the fluorescence intensity profiles of HADA, Ebp 1, BADA and Ebp 2 in each vertical plane along the longitudinal axis of mid‐division cells (Figure 3e). As expected, Ebp 1 and HADA fluorescence intensity profiles appeared at opposite hemispheres of each cell, where the maximum fluorescence intensity of HADA peaked at the newer hemisphere (0–75), while the maximum fluorescence intensity of Ebp 1 peaked at the older hemisphere (75–150). On the other hand, the fluorescence intensity profile of Ebp 2 peaked in both hemispheres and overlapped with the HADA fluorescence profile in the new hemisphere. The fluorescence intensity profile of BADA peaked at the septum and with minimal overlap with Ebp2. The overall fluorescence intensity of Ebp 2 was higher than Ebp 1, again supporting the results in Figure 1e that Ebp is continuously accumulating on the cell wall as the cell grows. These results confirmed our hypothesis that the cell wall must undergo maturation before Ebp exposure to the cell surface. We propose a model associating the processes of Ebp surface exposure and new cell wall synthesis during cell division such that new Ebp are surface exposed on the older cell wall in a sequential manner towards the cell pole to decorate the entire cell hemisphere (Figure 3f). Taken together, our results show that surface exposure of Ebp on the cell wall is dependent on the age of the Enterococcal cell wall and septal lipid II may not be necessary for anchoring of pili.

### 2.4 Pilus cell surface exposure location is independent of focally enriched septal sortases

**FIGURE 4:**
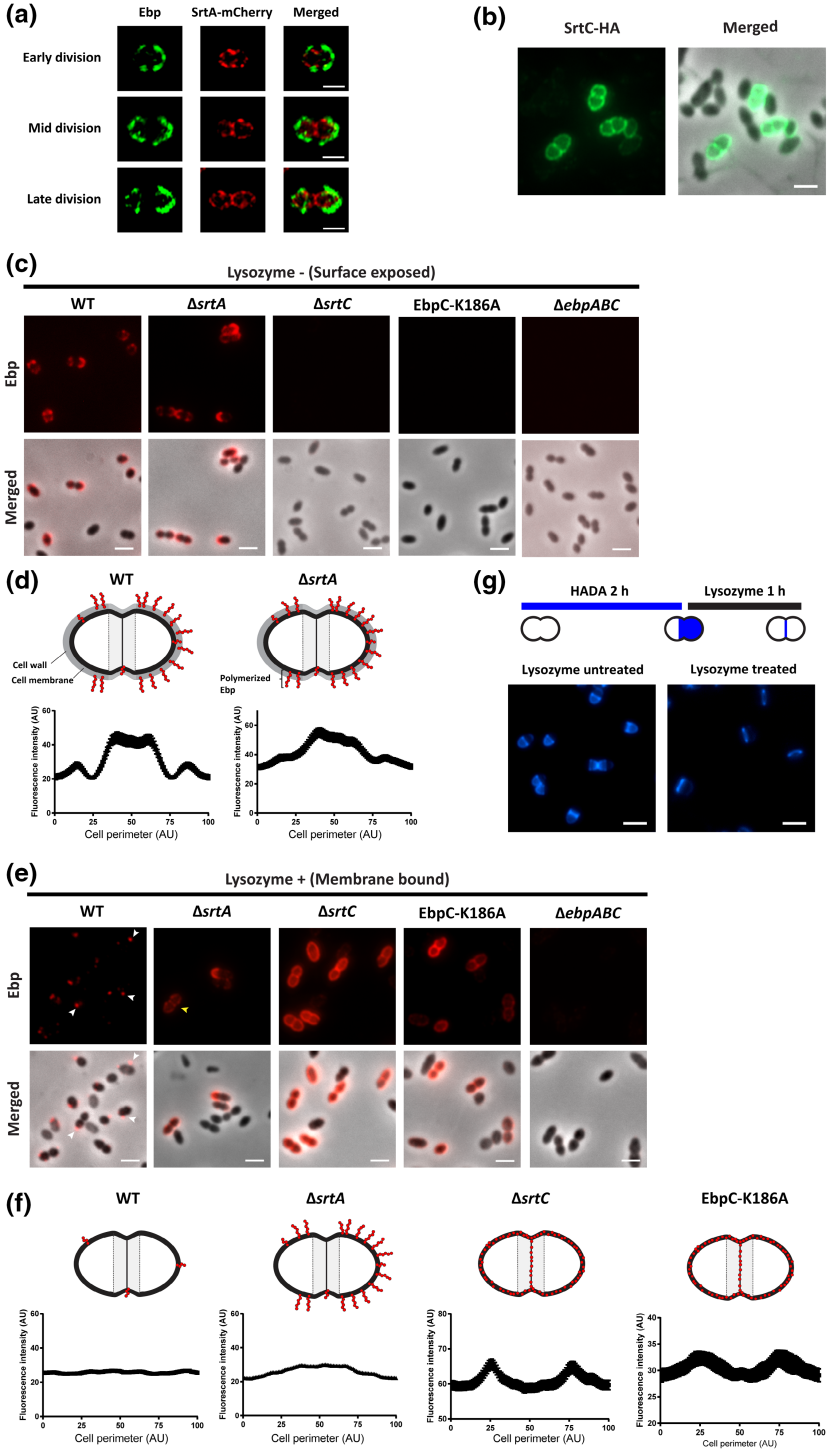
Sortases and Ebp co‐labelling and distribution of cell wall and membrane‐bound pili in *Enterococcus faecalis*. (a) Representative SrtA‐mCherry expressing cells, co‐labeled with Ebp and imaged by SIM. Scale bar: 1 μm. (b) Representative SrtC‐HA labeled cells imaged via epifluorescence. Scale bar: 2 μm. (c) Lysozyme untreated cells labeled for Ebp and imaged via epifluorescence and phase contrast microscopy. Scale bar: 2 μm. (g) Images of cells lysozyme treated cells before and after 2 h HADA labelling. Scale bar: 2 μm. (e) Lysozyme treated cells labeled for Ebp as described in Figure 2b. White arrows indicate stained Ebp on the surface of *E. faecalis* membranes in WT. Yellow arrow points to Ebp localized septally in Δ*srtA*. Scale bar: 2 μm. (d) and (f) depicts proposed Ebp positions and localization profiles of lysozyme treated (f) and untreated (g) cells.

Previously, we showed that SrtA was enriched at the *E. faecalis* cell septum (Kandaswamy et al., 2013; Kline et al., 2009). Yet in this study, we show that surface‐exposed Ebp, a SrtA substrate, is localized away from the cell septum and appear in a cell wall age‐dependent manner at the cell hemispheres. We postulated that, even though SrtA is enriched at the septum, peripheral SrtA may anchor Ebp to the hemispherical cell wall. To examine SrtA localization relative to Ebp, we labeled surface exposed Ebp in a *srtA*‐mCherry *E. faecalis* strain, enabling visualization of membrane‐anchored SrtA in cells with intact cell wall. Consistent with previous studies, we saw SrtA focally enriched at the septum and at puncta along the cell periphery (Figure 4a). SrtC, responsible for Ebp polymerization is similarly septum‐enriched and also found at foci at the cell periphery as previously reported (Kline et al., 2009) (Figure 4b). Ebp localization was coincident with peripheral SrtA puncta, rather than septal SrtA in all division phases (Figure 4a). This observation supports our data that Ebp becomes surface exposed at the cell hemispheres and may be anchored to the cell wall by peripheral SrtA. Polymerization of Ebp by SrtC occurs on the cell membrane, after which they are anchored to the cell wall by SrtA (Nielsen et al., 2013). To investigate if sites of SrtA‐dependent cell wall anchoring are only found at the cell hemispheres, we labeled Ebp in SrtA mutants, in which Ebp remain membrane‐bound and unattached to the cell wall. We predicted that if Ebp are attached to the cell wall at the hemispherical periphery in WT cells, they might similarly accumulate at the peripheral area of the membrane in a hemispherical pattern in Δ*srtA*. We first examined Ebp by immunolabeling EbpC prior to lysozyme treatment for cell wall removal. We observed hemispherical labelling of Ebp on the cell surface in Δ*srtA*, similar to WT (Figure 4c,d), which we verified to be membrane bound as previously described (Nielsen et al., 2013) (Figure S2). Together, these results tell us that despite polymerized pili being membrane bound when SrtA is absent, these pili can protrude out through the cell wall and be detected at the surface of the cell hemispheres.

After cell wall removal by lysozyme treatment, we expected to see no Ebp labelling on the protoplast of WT cells as Ebp anchored to the cell wall would have been removed. Strikingly, even though we no longer saw hemispherically localized Ebp, we observed cells decorated with Ebp foci at random locations on the protoplasts (Figure 4e, white arrows). In addition, the Ebp fluorescence intensity profile no longer showed high intensity at the positions corresponding to the hemisphere but instead, appeared flat (Figure 4f). The flat line suggests that the Ebp foci on the WT protoplasts are indeed randomly distributed along the cell perimeter, including the cell septum. On the other hand, membrane bound Ebp on Δ*srtA* protoplasts remained localized at the cell hemispheres. Notably, we also observed septal Ebp labelling in some Δ*srtA* protoplasts (Figure 4e, yellow arrows). No fluorescence was detected in Δ*ebpABC* protoplasts, indicating that the anti‐EbpC antibody was specific (Figure 4c,e). To ensure that the Ebp on the protoplast was not a result of incomplete cell wall removal, we performed the same lysozyme treatment on cells pre‐labeled with HADA for 2 h and observed complete removal of the peripheral cell wall (Figure 4g). We hypothesize that the Ebp foci observed on WT protoplasts could be sites of polymerization and membrane anchoring of pili prior to cell wall incorporation by SrtA. The presence of Ebp localized at the cell septum in WT protoplasts suggests that Ebp monomers might be secreted and polymerized at the septum and not yet exposed to the surface. Moreover, in lysozyme treated Δ*srtA*, apart from hemispherical labeled membrane bound Ebp, Ebp foci could also be found at the septal region of some cells (Figure 4f). The presence of Ebp septal labelling on Δ*srtA* protoplasts supports our speculation that newly polymerized pili may be buried under the septal cell wall occluding surface exposure. Taken together, these data show that the majority of surface‐exposed Ebp become cell wall anchored via SrtA and a subfraction remain membrane‐bound, perhaps transiently after membrane‐based pilus assembly and before SrtA mediated cell wall attachment. Coupled with the earlier observation that pilus exposure on the cell surface does not coincide with newly synthesized cell wall at the septum, we instead postulate that these surface exposed Ebp correlate with cell wall maturity mechanisms, consistent with studies in *S. aureus* showing that the mature PG is more porous due to modification by cell wall hydrolases (Pasquina‐Lemonche et al., 2020). Therefore, we postulate that the process of cell wall maturation in *E. faecalis* leads to increased porosity of the cell wall, thus resulting in increased surface exposure of pili.

### 2.5 Ebp polymerization is essential for hemispherical cell membrane localization

Our data thus far show that not all pili are cell wall anchored and a subset can be bound to the membrane. Moreover, these membrane‐bound pili can be surface exposed at the cell hemispheres even in the absence of SrtA. Here, the question arises of whether the hemispherical foci labeled with pilin antibodies represent polymerized Ebp or pilin monomers. Thus, to address if pilus polymerization is crucial for hemispherical localization of Ebp on the cell membrane prior to surface exposure, we examined the localization of Ebp monomers in Ebp polymerization deficient SrtC mutants to observe their cell wall and cell membrane localization. No pilin labeling was observed when Δ*srtC* cells were labeled without cell wall removal (Figure 4c) which was expected because membrane‐bound Ebp monomers are buried under the thick peptidoglycan layer. To image membrane bound Ebp monomers, we first removed the cell wall removal via lysozyme treatment followed by immunolabeling of SrtC‐deficient protoplasts for EbpC. In the absence of SrtC, we observed Ebp monomers localized throughout the cell protoplast and focally enriched at the cell septum, unlike membrane bound polymerized Ebp which are predominantly localized at the cell hemispheres (Figure 4e,f). To address the possibility that SrtC itself, independent of its function in pilus polymerization, is involved in hemispherical Ebp localization, we analyzed Ebp localization on Δ*ebpABC* p‐ebpABC^K186A^, a non‐polymerizing Ebp strain in which SrtC is still present (Nielsen et al., 2013). We performed the same EbpC immunolabeling assay as above and observed that even in the presence of SrtC, Ebp monomers in Δ*ebpABC* p‐ebpABC^K186A^ also became localized throughout the cell protoplast with focal enrichment at the septum (Figure 4e,f). These results suggest that SrtC mediated EbpC polymerization is essential for EbpC hemispherical localization. Furthermore, our findings hint that hemispherical localization of polymerized Ebp is a coordinated process independent of sortase localization but instead dependent on either cell maturation processes or other localization factors that might direct polymerized pili on the membrane to the hemispheres.

### 2.6 New pilus deposition at the cell hemisphere may be independent of new cell wall synthesis

**FIGURE 5:**
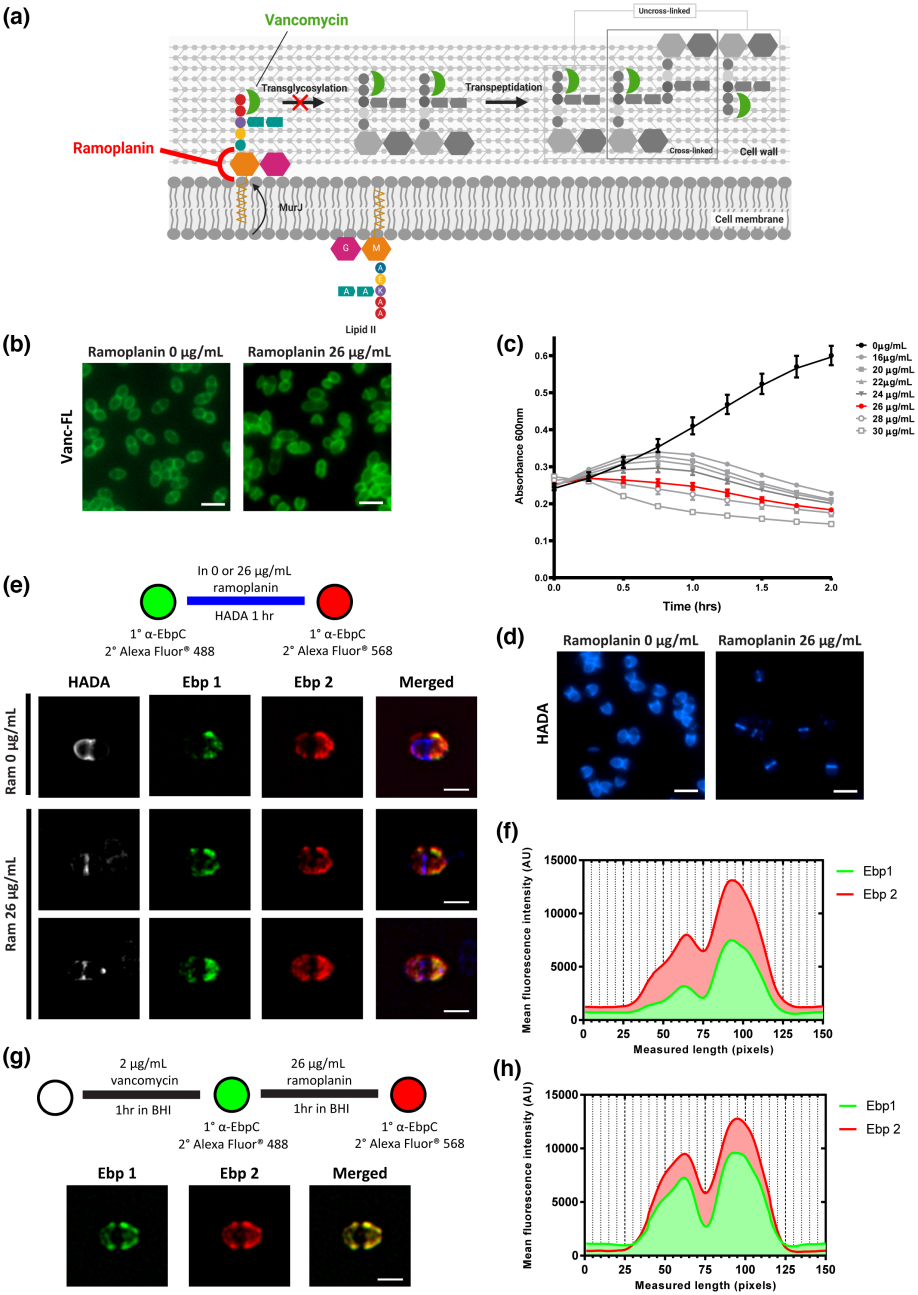
Cell wall synthesis inhibition by ramoplanin does not affect pili deposition at the cell hemispheres. (a) Schematic showing target sites of cell wall inhibiting drugs, ramoplanin and vancomycin. (b) Cells treated or untreated with ramoplanin were stained with BODIPY FL vancomycin and imaged. Scale bar: 2 μm. (c) Exponentially growing bacteria were treated with increasing concentrations of ramoplanin for 2 h at 37°C and measured at an absorbance of 600 nm. (d) HADA labelling of bacteria treated in the presence or absence of ramoplanin for 1 h. Scale bar: 2 μm (e) exponentially growing cells were harvested and labeled for Ebp in green (Ebp1) via immunofluorescence and grown in the presence or absence of ramoplanin with HADA for 1 h. A second Ebp labelling in red (Ebp 2) was performed and imaged by SIM. Scale bar: 1 μm. (f) Quantification of old and new Ebp fluorescence intensity along the longitudinal axis of ramoplanin‐treated cells from panel E from three independent experiments (*n* = 67). (g) Ebp chase labelling of cells pretreated with vancomycin followed by treatment with ramoplanin. Scale bar: 1 μm. (h) Quantification of old and new Ebp fluorescence intensity along the longitudinal axis of ramoplanin‐treated cells from (g) (*n* = 55). All experiments were performed at least 3 times.

Cell wall sorting machinery was originally characterized in *S. aureus* model whereby SrtA catalyzes the formation of a substrate‐lipid II intermediate which is eventually incorporated into the cell wall via transglycosylation and transpeptidation reactions (Perry et al., 2002). Our observations that new cell surface exposure of Ebp and formation of the Enterococcal cell wall do not occur simultaneously led us to postulate that Ebp may bypass precursor lipid II and instead be directly anchored onto the uncrosslinked cell wall by SrtA. The *E. faecalis* cell wall is about 50% crosslinked compared to 85% in *S. aureus* (Kim et al., 2015; Yang et al., 2017). This partial cell wall cross‐linking suggests that there is an abundance of free L‐Ala‐L‐Ala crossbridges in *E. faecalis* for anchoring of cell surface proteins. When *E. faecalis* was stained with BODIPY FL vancomycin (Vanc‐FL), a fluorescently labeled antibiotic that targets the D‐Ala‐D‐Ala residues in the cell wall (Figure 5a), we saw enrichment of fluorescence at the septum and staining at the cell periphery (Figure 5b). The intense fluorescence at the cell septum is indicative of D‐Ala‐D‐Ala on lipid II cell wall precursors, while the staining at the cell periphery indicates the D‐Ala‐D‐Ala on uncrosslinked cell wall. To test if Ebp deposition is dependent on cell wall synthesis, and hence the availability of lipid II precursors, we treated cells with ramoplanin to inhibit cell wall synthesis. Ramoplanin primarily targets lipid II precursors, sequestering them and rendering them unavailable for transglycosylation (Figure 5a), hence inhibiting new cell wall synthesis (Fang et al., 2006; Hu et al., 2003). We monitored growth in increasing concentrations of ramoplanin and determined the bacteriostatic concentration to be 26 μg ml^−1^ for *E. faecalis* (Figure 5c). To verify that cell wall synthesis was inhibited at this concentration, we grew ramoplanin‐treated cells together with HADA. After 1 h of incubation, HADA was only detected at the cell septum (Figure 5d), unlike the greater hemispherical localization in ramoplanin untreated cells (Figure 2b). These findings indicate that cell wall synthesis was halted after pre‐existing PG chains were cross‐linked by transpeptidases at the cell septum, before lipid II was sequestered by ramoplanin, thus preventing formation of PG chains by transglycosylation. To determine if new Ebp was deposited onto the cell surface when cell wall synthesis was inhibited, we performed the HADA‐Ebp chase experiment in the presence and absence of ramoplanin. Interestingly, at bacteriostatic concentrations of ramoplanin, we saw new Ebp deposition at the cell hemisphere despite cell wall synthesis inhibition (Figure 5e). We validated this observation by quantifying the fluorescence intensity of old (Ebp 1) and new Ebp (Ebp 2) along the length of the cell (Figure 5f). Indeed, there was an increase in fluorescence intensity along both cell hemispheres for Ebp 2 as compared to Ebp 1, especially in the “new” cell hemisphere (25 to 75). The increase in Ebp 2 fluorescence signal at the cell hemispheres shows that new Ebp are still being deposited on the cell wall, independent of new cell wall synthesis. Given the partial crosslinking of the *E. faecalis* cell wall (Kim et al., 2014; Yang et al., 2017), we propose that Ebp can bypass the cell wall synthesis machinery to be anchored by SrtA onto uncrosslinked cell wall. To eliminate the possibility that new surface‐exposed Ebp in ramoplanin treated cells were membrane‐bound, we performed western blot to examine presence of the three subunits of Ebp in both the cell wall and protoplast fractions of both ramoplanin treated and untreated cells. Indeed, the high molecular weight (HMW) ladder, representing polymerized pili, visible on the Ebp immunoblots were present only in the cell wall fraction in ramoplanin treated cells (Figure S3), demonstrating that the new Ebp were being incorporated into the cell wall via uncrosslinked PG away from the septum. Accordingly, when we stained ramoplanin treated cells with Vanc‐FL, we observed homogeneous cell wall staining throughout the cell and lack of fluorescence enrichment at the septum, indicative of an abundance of uncrosslinked PG in the cell and inhibition of new cell wall synthesis, respectively (Figure 5b). To further investigate if Ebp can be anchored to uncrosslinked cell wall, we blocked uncrosslinked cell wall by pretreating cells with vancomycin at MIC of 2 μg ml^−1^ (Diaz et al., 2012; Kristich et al., 2011) for one hour in BHI and performed EbpC chase experiment in the presence of ramoplanin. Pretreatment with vancomycin should block uncrosslinked cell wall and should prevent new Ebp deposition on the cell wall, if our model is correct. Indeed, we did not observe new Ebp deposition in vancomycin and ramoplanin treated cells (Figure 5g) and Ebp quantification showed similar fluorescence intensity profiles for both Ebp1 and Ebp2 (Figure 5h). In contrast to the current paradigm for sortase substrate incorporation into the cell wall, our data suggest that *E. faecalis* Ebp can be anchored onto uncrosslinked cell wall, which facilitates exposure to the cell exterior. We propose a new paradigm for *E. faecalis* Ebp deposition where SrtA mediated Ebp attachment of pili bypasses the need for lipid II‐substrate intermediates and directly anchors pili onto uncrosslinked PG.

## 3 DISCUSSION

Spatial localization of cell wall‐anchored surface proteins has been studied in various species of Gram‐positive cocci. In *S. aureus* and *Streptococcus pyogenes*, cell‐wall associated proteins are localized at the septal region or at the poles depending on the presence or absence of a conserved YSIRK motif within substrate signal sequences, respectively (Carlsson et al., 2006; Raz et al., 2012). In *S. pneumoniae*, sortase‐assembled pili are distributed in a non‐homogenous manner, focally localized at the cell surface at discrete puncta (Fälker et al., 2008). In *E. faecium*, two distinct types of sortase‐assembled pili are expressed, namely PilA and PilB. PilA are evenly distributed around the cell, while polymerized PilB are observed at the older pole at exponential phase (Hendrickx et al., 2008, 2010). Another example of polar distribution of sortase anchored pili are PilB in *Streptococcus agalactiae* (Brega et al., 2013). Hence, pilus localization in Gram‐positive cocci is determined in both a spatial and temporal manner.

Here, we show that surface‐exposed *E. faecalis* Ebp are distributed in an asymmetrical manner such that the older hemisphere is saturated with Ebp while new Ebp becomes surface exposed at the newer hemisphere. This heterogeneous localization pattern has been reported for other *E. faecalis* surface‐exposed virulence factors such as aggregation substance (AS) (Olmsted et al., 1993; Wanner et al., 1989), but this is the first study showing *how* the hemispherical localization of SrtA substrates takes place over time. Although localization of surface proteins has been explored in *E. faecalis*, the spatiotemporal distribution of these proteins has not been characterized prior to this study. Using time‐lapse microscopy, we traced the movement of labeled pili along the cell hemisphere and showed that they remain at a fixed position on the cell wall. While positionally fixed on the cell wall, labeled pili are subsequently pushed apart by new cell wall material synthesized at the septum. Ebp turnover appears to be low as labeled pili remain intact throughout cell division. We addressed Ebp turnover by performing a double Ebp immunofluorescence chase experiment using different fluorescently labeled antibodies and observed that new pili are deposited and/or become surface‐exposed temporally towards the poles and eventually, the deposited pili converge over the whole cell hemisphere as the pole ages. Hence pilus and AS deposition in *E. faecalis* are coordinated in a specific spatiotemporal manner.

The appearance of septum‐excluded pili on the cell is associated with the age of the cell wall. When different FDAA labels were successively pulsed in short (5 min) or long (40 min) waves in *E. faecalis*, there was little to no overlap between each label that was pulsed, suggesting there is minimal cell wall turnover, similar to *S. pneumoniae* (Boersma et al., 2015). This finding contrasts with *Bacillus subtilis*, where rapid cell wall turnover is observed when similar FDAA labelling experiments were performed (Boersma et al., 2015; Kuru et al., 2015). The minimal cell wall turnover trait in *E. faecalis* supports the observation that pre‐labeled pili remain locked in position as the cell continues to grow and divide. Long pulse chase of FDAA PG labelling in *E. faecalis* showed distinct hemispherical labelling of the older and newer cell wall, indicating that one hemisphere of the cell is always older than the other, as would be expected. This hemispherical labelling pattern is reflective of Ebp localization where surface‐exposed Ebp is saturated at one hemisphere of the cell. Together, these similarities led us to speculate that new pili could be intercalated with newly synthesized cell wall. However, our Ebp and cell wall co‐staining results showed that previous studies do not fit with the present model. Instead, the labelling of new PG and surface‐exposed pili was almost mutually exclusive, and there was little overlap between the two. When a second Ebp staining was performed after growth in fresh media, new pili appeared at the older pre‐labeled cell wall. This result was unexpected because the current paradigm favors that SrtA anchors its substrates onto the septal cell wall precursor, lipid II, which is subsequently incorporated onto the cell wall via transglycosylation and transpeptidation (Ton‐That et al., 1997; Ton‐That & Schneewind, 1999). The lack of spatial or temporal coordination between newly synthesized PG and surface‐exposed pili suggest that pilus deposition can be independent of lipid II precursors, and that pili can be deposited via an alternative mechanism.

Several lines of evidence from studies in *S. aureus* indicate that lipid II serves as the cell wall substrate for SrtA. Antibiotic treatment targeting transpeptidation did not affect surface protein anchoring in *S. aureus* (Ton‐That & Schneewind, 1999). Furthermore, a mature assembled cell wall was not required for the cleavage of surface protein precursors in staphylococcal protoplasts (Ton‐That & Schneewind, 1999). The results of our experiments with *E. faecalis* showed that inhibition of cell wall synthesis with ramoplanin, an antibiotic targeting lipid II, did not affect pilus deposition. We observed new pili appearing at the cell hemispheres and not at the septum where new cell wall synthesis takes place. However, when cells were pre‐treated with vancomycin to block SrtA access to uncrosslinked cell wall, we observed no new pilus deposition at the cell hemisphere. Based on these observations, we suggest that SrtA in *E. faecalis* can anchor cell wall proteins directly onto the crossbridge of uncross‐linked, older cell wall. Compared to *S. aureus*, which has approximately 85% of crosslinked PG, the percentage of crosslinked cell wall in *E. faecalis* is lower at approximately 50% (Kim et al., 2014; Yang et al., 2017). The lower percentage of PG crosslinking in *E. faecalis* is attributed to the shorter PG crossbridge of only 2 L‐Ala‐L‐Ala compared to the pentaglycine crossbridge in *S. aureus* (Yang et al., 2017). The abundance of uncrosslinked PG in *E. faecalis* may serve as anchor points for SrtA to anchor polymerized pili.

But there is a conundrum: we see SrtA and SrtC both at the septum and at the periphery, but only see surface‐exposed polymerized Ebp at the periphery. We propose at least two possible explanations for this observation. (1) Pili are attached to the cell wall at the septum, but they remain inaccessible to antibodies until a later point in the cell cycle or in cell wall maturity. (2) Pili are only anchored to hemispherical cell wall, and septal SrtA are inactive for pilus anchoring. These possibilities are not mutually exclusive. In support of the first explanation, in both lysozyme treated WT and sortase mutant cells in which the cell walls are removed, we observe Ebp puncta on the peripheral membrane of both hemispheres, as well as at the septum. Why then do we not see surface exposed pili at the septum? This absence of pili could be because the cell wall is more dense at the septum and more porous at the periphery in an age dependent manner due to cell wall modifications conferred by cell wall hydrolases (Wheeler et al., 2015), leading to more surface‐exposed pili on the older cell wall. In support of the second explanation, we observe saturation of Ebp at the cell hemispheres of sortase mutant protoplasts, even when they are only membrane bound. Although we observe Ebp puncta at the septal membrane, it is possible that Ebp are polymerized at the septum, possibly within punctate microdomains, and subsequently move within the cell membrane towards the older cell hemisphere for anchoring by SrtA. Microdomains in the bacterial cell membrane are dynamic and fluidic in nature (Los & Murata, 2004; Miller et al., 2019). The accumulation of membrane bound pili at the hemispheres of Δ*srtA* suggests that the sites of protein cell wall anchoring is at the hemipsheres. What is not known is whether there is any regulation or coordination involved in guiding membrane bound pili to the hemispheres.

Future work to fully understand how *E. faecalis* Ebp assembly, cell wall anchoring, and surface‐exposure is coordinated should include the contribution of cell wall hydrolases. Ebp surface exposure in an *atlA* mutant strain deleted for the major *E. faecalis* cell wall hydrolase (Eckert et al., 2006) occurs at the hemispheres, similar to WT (data not shown). There are at least eight cell wall modifying enzymes encoded in the *E. faecalis* genome (Arthur et al., 1994; Benachour et al., 2012; de Roca et al., 2010; Emirian et al., 2009; Kurushima et al., 2015; Mesnage et al., 2008), and so it would be of interest to determine which (or which combination) might contribute to cell wall porosity, as shown in *B. subtilis* and *S*. aureus (Pasquina‐Lemonche et al., 2020) or turnover in *E. faecalis*, leading to Ebp appearance on the old wall. In addition, or alternatively, other cell modifications such as teichoic acid incorporation or modification (e.g. by alanylation) may be enriched in the more mature PG and may favor SrtA substrate incorporation, as has been recently demonstrated in *S. aureus* (Zhang et al., 2021). It is also possible that even though SrtA is localized at the septum, the enzymes may be less active for transpeptidation than peripheral SrtA. In *S. aureus*, it has been proposed that the dimeric form of SrtA is more active than the monomeric enzyme (Lu et al., 2007), so perhaps *E. faecalis* SrtA dimerization is favored within the peripheral membrane.

**FIGURE 6:**
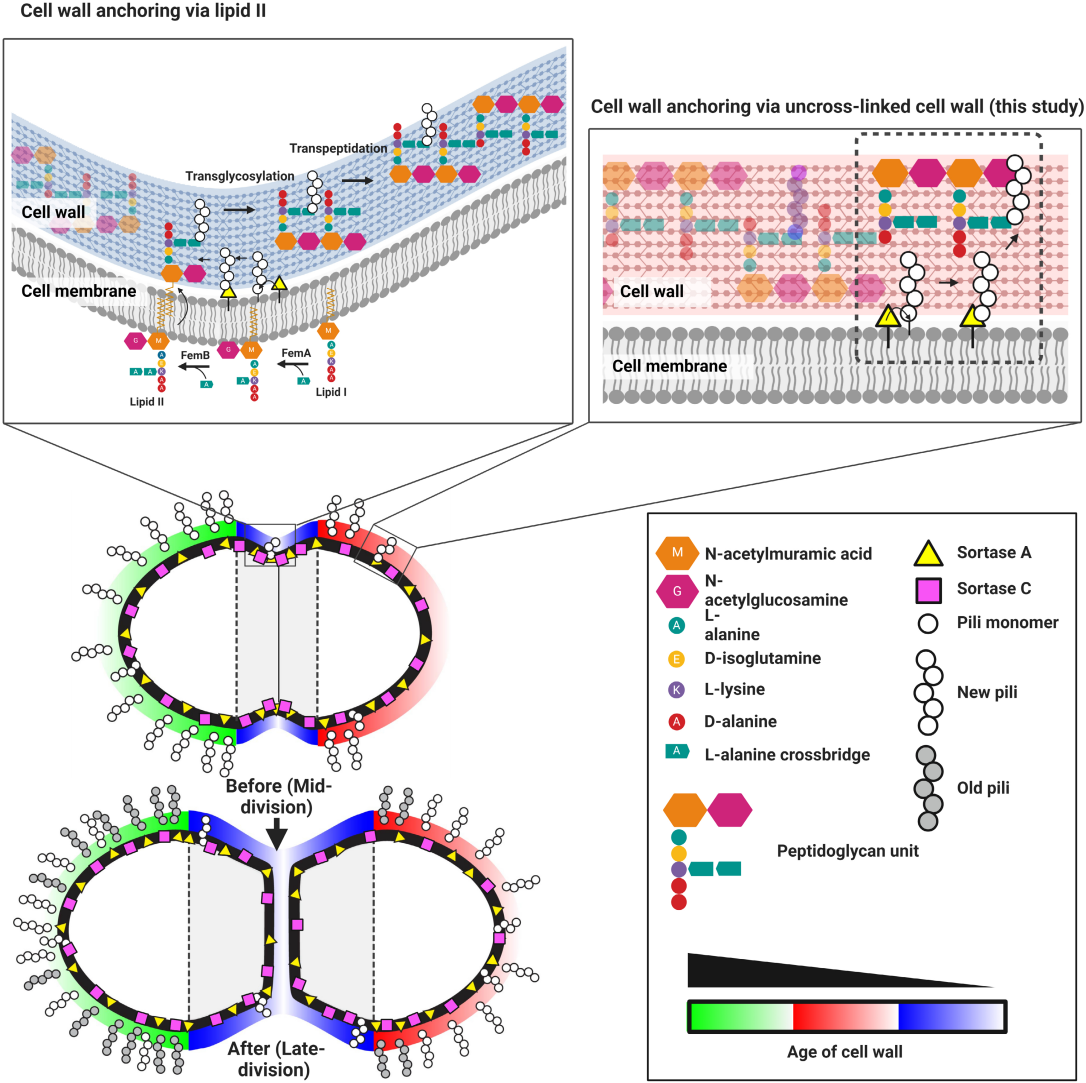
Spatialtemporal models of *Enterococcus faecalis* pili cell wall anchoring and pili deposition. Polymerized pili can either be anchored onto the cell wall via lipid II at the cell septum or uncrosslinked cell wall at the cell periphery. Sortases are localized along the cell membrane but not all are active at the same time. Surface‐exposed polymerized pili are localized asymmetrically where the older hemisphere is more saturated with pili. Over time, the cell elongates and newer pili are deposited in a chronological manner towards the pole, eventually populating the whole cell hemisphere.

Taken together, we propose a model where the older hemisphere of *E. faecalis* will always be more saturated with surface‐exposed polymerized pili than the younger hemisphere, where newer pili are just beginning to be exposed to the surface. As the cell grows and elongates during division, new pili are anchored onto uncrosslinked cell wall at the cell hemispheres in sequential order towards the pole and eventually saturate the whole hemisphere (Figure 6). A limitation of this study is that there is no direct evidence showing anchoring of the pilin subunits on mature peptidoglycan rather than lipid II, which we hope future studies will be able to address. This study provides an in‐depth characterization of the spatiotemporal dynamics of peptidoglycan and surface‐exposed virulence factors in *E. faecalis* and highlights the possibility of an alternative route to cell wall protein anchoring by SrtA in which substrates are preferentially attached to mature uncrosslinked cell wall rather than lipid II cell wall precursors.

## 4 EXPERIMENTAL PROCEDURES

### 4.1 Bacterial culture and strains

Bacterial strains and plasmids used in this study are listed in Table S1. Unless otherwise stated, *E. faecalis* strains were streaked onto brain heart infusion (BHI) agar from 25% glycerol stocks stored at −80°C and grown overnight at 37°C. Single colonies were then inoculated into BHI broth (BD Difco, USA) and grown overnight statically for 16 to 18 h at 37°C. To obtain cells at mid log phase, the overnight cultures were subcultured at 1:10 dilution into fresh BHI media and grown to OD_600_ 0.5 ± 0.05. Cells were then normalized to OD_600_ 0.5 in 1 ml 0.1 M phosphate buffer (PB) for subsequent use unless otherwise stated. Where appropriate, antibiotics were added at the following concentrations: tetracycline (Tet), 15 μg ml^−1^; Kanamycin (Kan), 500 μg ml^−1^.

### 4.2 Genetic manipulation

*E. faecalis* OG1RFΔ*srtA* was transformed with plasmid pGCP123::P*srtA srtA‐2 L‐mCherry* in which SrtA‐mCherry fusion is expressed on a plasmid and transcribed from the native *srtA* promoter. The wild‐type *srtA* native promoter was amplified using primers KpnI‐PsrtA‐F (5′‐ATCCGGTACCGCTTGTTTCTTTTACTTTAAAATTCCA‐3′) and XhoI‐PsrtA‐R (5′‐AAGCCTCGAGATTCTCCCTCCTTTTAATGT‐3′) and the *srtA* gene was amplified using primers XhoI‐SrtA‐F (5′‐GAATCTCGAGATGCGCCCAAAAGAGAAAAA‐3′) and EcoRI‐SrtA‐R (5′‐ATCCGAATTCAGCCACCCAATCGGCTAA3′), using *E. faecalis* OG1RF as template. mCherry was amplified using primers EcoRI‐SrtA‐R (5′‐ATCCGAATTCATGGTGAGCAAGGGC‐3′) and NotI‐STOP‐XbaI‐BamHI‐mCherry‐R (5′‐AATCGCGGCCGCCTATCTAGAGGATCCCTTGTACAGCTCGTCCAT‐3′). These three PCR products were ligated together using primer embedded restriction sites XhoI and EcoRI respectively. The resulting fusion product was cloned into PGCP123 (Nielsen et al., 2012) using primer embedded restriction sites KpnI and NotI. The expression and stability of the fusion was verified with anti‐mCherry (Invitrogen, USA) and anti‐SrtA (SABio, Singapore) immunoblotting of whole‐cell *E. faecalis* lysates.

### 4.3 Immunofluorescence microscopy

Immunolabeling of cells was performed as described in (Kandaswamy et al., 2013) with modifications. Mid log phase cells were normalized to OD_600_ 0.5 and washed once in 0.01 M low salt phosphate buffer (PB). Cells that do not require lysozyme treatment were immediately blocked after washing. When cell wall removal was required, cells were fixed in 4% (wt/vol) paraformaldehyde (PFA) and incubated for 20 min at room temperature (RT) to improve the rigidness of the cell. For cell wall removal, cells were treated with 10 mg ml^−1^ lysozyme for 37°C for 1 h to expose the membrane‐bound proteins before blocking. Blocking was performed by incubating the cells in 2% (wt/vol) bovine serum albumin (BSA) in PB for 20 min at RT. For Ebp or SrtC staining, cells were incubated with guinea pig anti‐EbpC serum (Afonina et al., 2018) or rabbit anti‐HA antibody (H6908; Sigma Aldrich) at 1:500 dilution in PB‐2% BSA for 1 h at RT. Next, cells were washed once in PB and incubated with fluorescent‐conjugated secondary antibody at 1:500 dilution in PB‐2% BSA for 1 h (Alexa Fluor 405/488/568–goat anti‐guinea pig antibody for EbpC or goat anti‐rabbit antibody for SrtC‐HA [Invitrogen, Inc., USA]). Cells were then washed once in PB and resuspended in 1 ml PB. Before sample mounting, microscope glass slides were washed once in filtered 70% ethanol followed by filtered ultrapure water (18.2 ohm) and dried. Hydrophobic wells measuring 1.25 cm in diameter were drawn on the glass slide using a PAP pen (Sigma Aldrich, Singapore). 20 μl of cell suspension was spotted onto each well and allowed to dry in a 60°C oven. Samples were covered with 5 μl of mounting media (Vectashield®, USA) and sealed with glass coverslips. Widefield microscopy was performed using an inverted epi‐fluorescence microscope (Zeiss Axio observer Z1, Germany) fitted with a Plan‐Neofluar 100x/1.3 oil Ph3 objective lens using ZEN 2 (blue edition) software. The images were acquired using AF568/cy3 filter cube sets fitted with a 530–580 nm bandpass excitation filter and a 585 nm‐long pass barrier filter. For unbiased image analysis, exposure times were fixed for all experiments. Images were processed using FIJI (Schindelin et al., 2012) and Adobe Photoshop CS 5.1.

### 4.4 Super‐resolution structured illumination microscopy (SIM)

All SIM imaging were performed using an alpha Plan‐Apochromat 100x/1.46 oil DICIII objective lens and pco.edge sCMOS camera fitted onto an Elyra PS.1 microscope (Zeiss). Laser wavelengths of 561, 488 and 405 nm at 20% power were used to excite red, green and blue fluorescent probes respectively. Images were acquired using five grid rotations with 51 μm grating period and reconstructed using Zeiss software (ZEN 2012 SP5 FP2, black edition). Images were processed using FIJI (Schindelin et al., 2012) and Adobe Photoshop CS 5.1.

### 4.5 Quantitative analysis of Ebp fluorescence distribution on single cells

For wide field images, fluorescence distribution of Ebp staining were quantified as described previously (Chilambi et al., 2020; Kandaswamy et al., 2013). Briefly, mid log phase cells (1.5 μm −2 μm in length) were first detected using a MATLAB function, Projected System of Internal Coordinates from Interpolated Contours (PSICIC) (Guberman et al., 2008). Cell perimeters of each cell were traced and given an arbitrary unit from 0 to 100 where positions 0, 100 and 50 mark the poles of the cell while positions 25 and 75 mark the division septum of the cell. The orientation profiles of cells were manually arranged such that 0 always corresponds to the pole with lower fluorescence intensity. A minimum of 70 cells were quantified for each analysis. Fluorescence intensity values corresponding to each position on the cell were plotted to generate a fluorescence distribution pattern.

For quantification of SIM images, at least ten individual cells were cropped and orientated along the longitudinal cell axis during such that the new cell hemisphere is always on the left. Fluorescence intensity values were averaged across the vertical plane of the cell and plotted against the length of the cell using FIJI (Schindelin et al., 2012). Fluorescence intensity plots of each fluorescence channel were obtained for each cell, compiled, and averaged to obtain the final plot.

### 4.6 Fluorescent D‐amino acid (FDAA) labelling

Cells were subcultured 1:10 in 5 ml BHI and grown to early log phase (OD_600_ 0.25 ± 0.05). Cells were normalized to OD_600_ 0.25 in pre‐warmed BHI and the FDAA, 7‐hydroxycoumarin‐3‐carboxylic acid 3‐amino–D‐alanine (HADA) (Kuru et al., 2015), was added to a final concentration of 250 μM. Cells were then grown for either 5, 20, 40 or 120 min at 37°C with agitation. To halt the labelling, cells were placed on ice and washed three times in ice cold PB. Cells were then mounted onto clean glass slides before imaging by SIM. For FDAA sequential labelling, cells were labeled first with the green derivative of BODIPY‐FL 3‐amino‐D‐alanine (BADA) for 5 min or 40 min at 37°C in BHI, washed once in ice cold PBS and then labeled with the red FDAA derivative, TAMRA 3‐amino‐D‐alanine (TADA) for 5 min or 40 min at 37°C in BHI. Cells were then washed once in ice cold PBS and incubated in BHI with HADA for 10 min or 20 min at 37°C, washed once in PBS and mounted onto glass slides and imaged via SIM as described above.

### 4.7 Time lapse imaging

BHI agarose (1%) gel pads were prepared by sandwiching molten BHI agarose between two glass slides. Once solidified, 1 cm by 1 cm agarose gel squares were cut out. Cells were harvested at mid‐log phase and immuno‐labeled for EbpC as described above. Next, 5 μl of cells were spotted and evenly spread across the agarose gel pad and sealed with a glass cover slip using paraffin wax. Images were then taken every 15 min at room temperature for 2.5 h using an inverted epi‐fluorescence microscope (Zeiss Axio observer Z1, Germany) fitted with a 100x/1.3 oil Ph3 objective lens.

### 4.8 Triple Ebp chase labeling

*E. faecalis* OG1RF cells were grown in BHI to mid log phase before being subjected to three rounds if EbpC staining via immunofluorescence. Cells were first stained with a green fluorescent secondary antibody, Alexa Fluor 488 (Invitrogen, Singapore). After the first EbpC labeling, cells were washed and grown in BHI for 1 h at 37°C, and stained for EbpC with a blue fluorescent secondary antibody, Alexa Fluor 405. Lastly, cells were washed, grown again in BHI for 1 h at 37°C and similarly labeled for EbpC but with a red fluorescent secondary antibody, Alexa Fluor 568. After the final staining, cells were mounted and imaged via SIM.

### 4.9 Determination of bacteriostatic concentration of ramoplanin

*E. faecalis c*ells were grown to OD 0.4 at 37°C and subsequently transferred into a 96‐well plate containing ramoplanin with the respective concentrations (0, 16–30 μg ml^−1^) in BHI. Cells were incubated at 37°C for 2 h and absorbance (600 nm) was read every 15 min using a Tecan M200 microtiter plate reader. The bacteriostatic concentration of ramoplanin was determined as the lowest dose of antibiotics that showed a decrease in absorbance reading within the first hour of incubation.

### 4.10 Ramoplanin Ebp‐HADA chase experiment

*E. faecalis* cells were grown to mid log phase, followed by EbpC labelling using a green fluorescent secondary antibody, Alexa Fluor 488. After washing once in PB, cells were then grown in the presence or absence of 26 μg ml^−1^ ramoplanin with 250 μM HADA in BHI at 37°C for 1 h. Lastly, cells were washed and labeled for EbpC, instead with a red fluorescent secondary antibody, Alexa Fluor 568. SIM imaging and quantification of EbpC fluorescence intensity profiles were performed as described above.

### 4.11 Vancomycin and ramoplanin Ebp chase experiment

Overnight *E. faecalis* cells were subcultured at 1:10 and grown to early log phase at OD 0.2 before addition of vancomycin at MIC of 2 μg ml^−1^ for 1 h in BHI. Cells were then immuno‐labeled for EbpC using using a green fluorescent secondary antibody, Alexa Fluor 488. After washing once in PB, cells were grown in the presence of 26 μg ml^−1^ ramoplanin at 37°C for 1 h. Lastly, cells were washed and labeled for EbpC, with a red fluorescent secondary antibody, Alexa Fluor 568. SIM imaging and quantification was performed as described above.

## AUTHOR CONTRIBUTIONS

**Pei Yi Choo:** Data curation; formal analysis; methodology; validation; visualization; writing – original draft; writing – review and editing. **Charles Y. Wang:** Resources. **Michael S. VanNieuwenhze:** Resources. **Kimberly A. Kline:** Conceptualization; supervision; writing – original draft; writing – review and editing.

## CONFLICT OF INTEREST

The authors declare no conflict of interest.

## Supporting information


**Appendix S1:** Supporting Information

## Data Availability

Data available on request from the authors.
