# Supplementary material for: Spatial and temporal localization of cell wall associated pili in Enterococcus faecalis
Source: Mol Microbiol. 2022 Dec 7;119(1):1–18. doi: 10.1111/mmi.15008 (PMC10107303; doi:10.1111/mmi.15008)
Supplement: Supplementary file 1 — Appendix S1: Supporting Information [file MMI-119-1-s001.docx]

**Supplementary material**

**SI Experimental Procedures**

***Aggregation substance chase labelling***

*E. faecalis* OG1RF pCF10 cells were grown overnight in BHI supplemented with 15 μg/mL tetracycline and diluted 1:10 in 5mL BHI in the presence of fresh media. To induce aggregation substance (AS) expression, 0.12ng/mL of cCF10 peptide was added for 30 minutes at 37°C, shaking at 200 rpm. Cells were then washed once before the first immunofluorescence staining was performed as described previously using rabbit anti-AS serum primary antibody and Alexa fluor 488-goat anti-rabbit secondary antibody (Thermo Fisher Scientific, USA). To induce a second round of AS expression, cells were washed and allowed to grow in fresh BHI supplemented with additional 0.12ng/mL cCF10 peptide for the second round of AS induction. Following the second incubation of cCF10, the second AS immunofluorescence labelling was performed as described with Alexa Fluor 568-goat anti-rabbit secondary antibody (Thermo Fisher Scientific, USA) instead. Cells were then mounted onto glass slides and imaged by SIM.

***Western blot***

Cells were harvested and normalized to OD 0.5 before spinning down at 14 000 rpm for 2 mins. The supernatants were collected to perform Trichloroacetic acid (TCA) precipitation of proteins while the cell pellets were washed once in PBS and.-incubated with 10 mg/mL lysozyme in a 37°C water bath for 1 hr. TCA precipitation of supernatant was performed by incubating the supernatant with 25% TCA for 10 mins at 4°C, spinning down at 14 000 rpm, followed by washing in ice cold acetone. Cell wall and protoplast fractions were separated by centrifugation at 14 000 rpm for 5 minutes, where the supernatant will make up the cell wall fraction and pellet will make up the protoplast fraction. Both protoplast and supernatant fractions were resuspended in 100 μL of PBS. For whole cell lysate fractions, no centrifugation was performed after lysozyme incubation. NuPAGE™ LDS sample buffer (Thermo Fisher Scientific, USA) was added to all fractions before boiling for 20 minutes. Ebp and SecA blots were run on NuPAGE 3 to 8% Tris- acetate and NuPAGE 10% Bis-Tris pre-cast gels respectively for 50 minutes at 120 V in Tris-acetate buffer. Proteins were subsequently transferred onto a PVDF membrane using an iblot™ gel transfer machine and iblot™ transfer stacks (Thermo Fisher Scientific, USA). Membranes were then blocked in 0.1% v/v Tween- 5% bovine serum albumin-PBS (Sigma Aldrich, Singapore) overnight at 4°C shaking. The next day, blocking buffer was removed and replaced with primary antibody (rabbit anti-EbpA, rabbit anti-EbpB, guinea pig anti-EbpC and rabbit anti-SecA) at a dilution of 1:3000 for 1 hour at RT and washed three times with 0.01% Tween-PBS. Membranes were then incubated in secondary antibody (IgG guinea pig or rabbit conjugated horseradish peroxidase) (Thermofisher Scientific, Singapore) at a dilution of 1:6000 for 1 hour and washed three times in 0.01% Tween-PBS. Protein bands were detected by chemiluminecence using SuperSigmal™ west femto maximum sensitivity substrate kit (Thermofisher Scientific, USA).

**
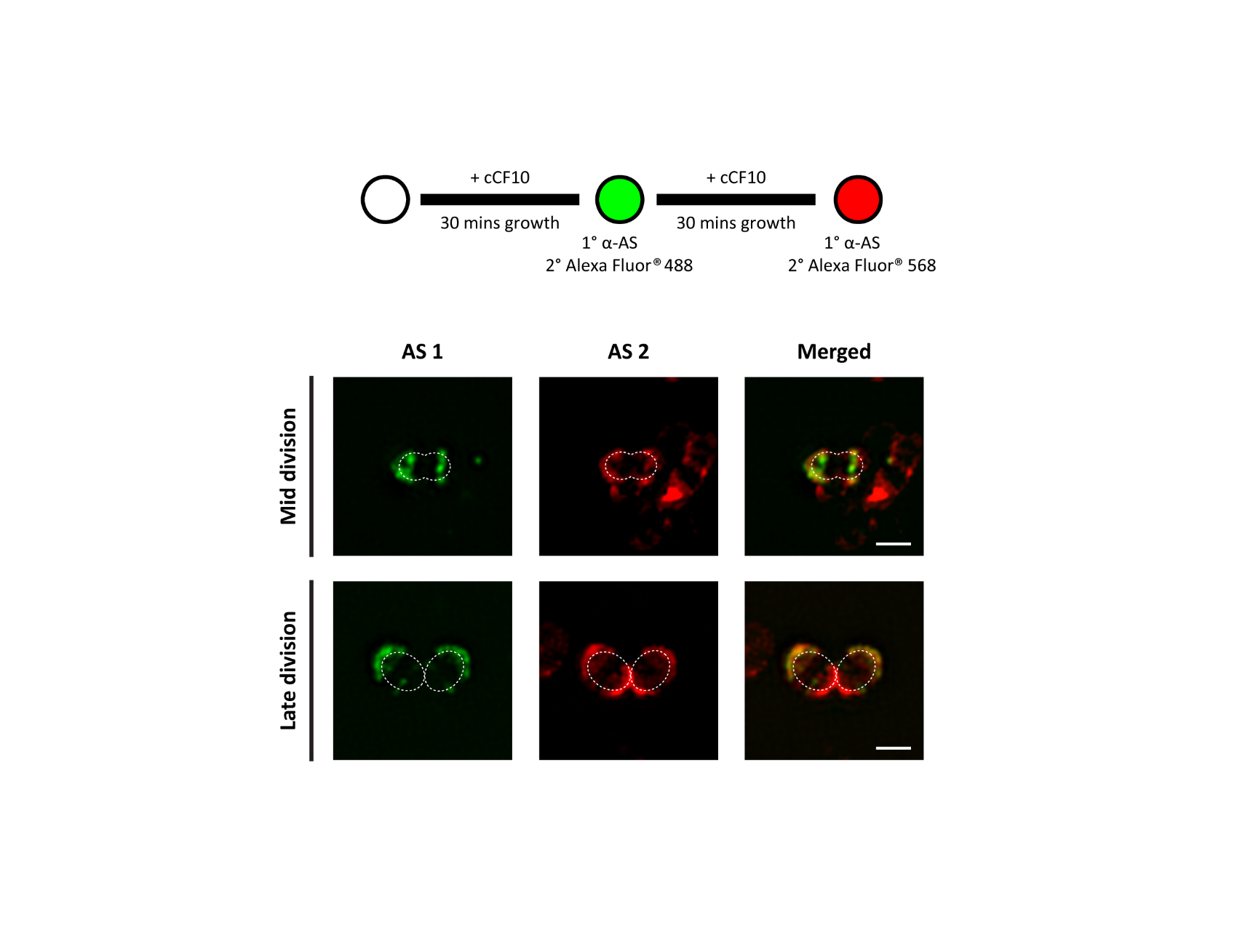
**

**Fig S1. Aggregation substance has similar distribution pattern as Ebp.** Chase labelling of AS via immunofluorescence at 30 minutes post induction with cCF10 peptide using green and red fluorescent conjugated secondary antibodies as shown in the schematic where. Representative mid and late division phase cells are shown. Scale bar: 1 µm


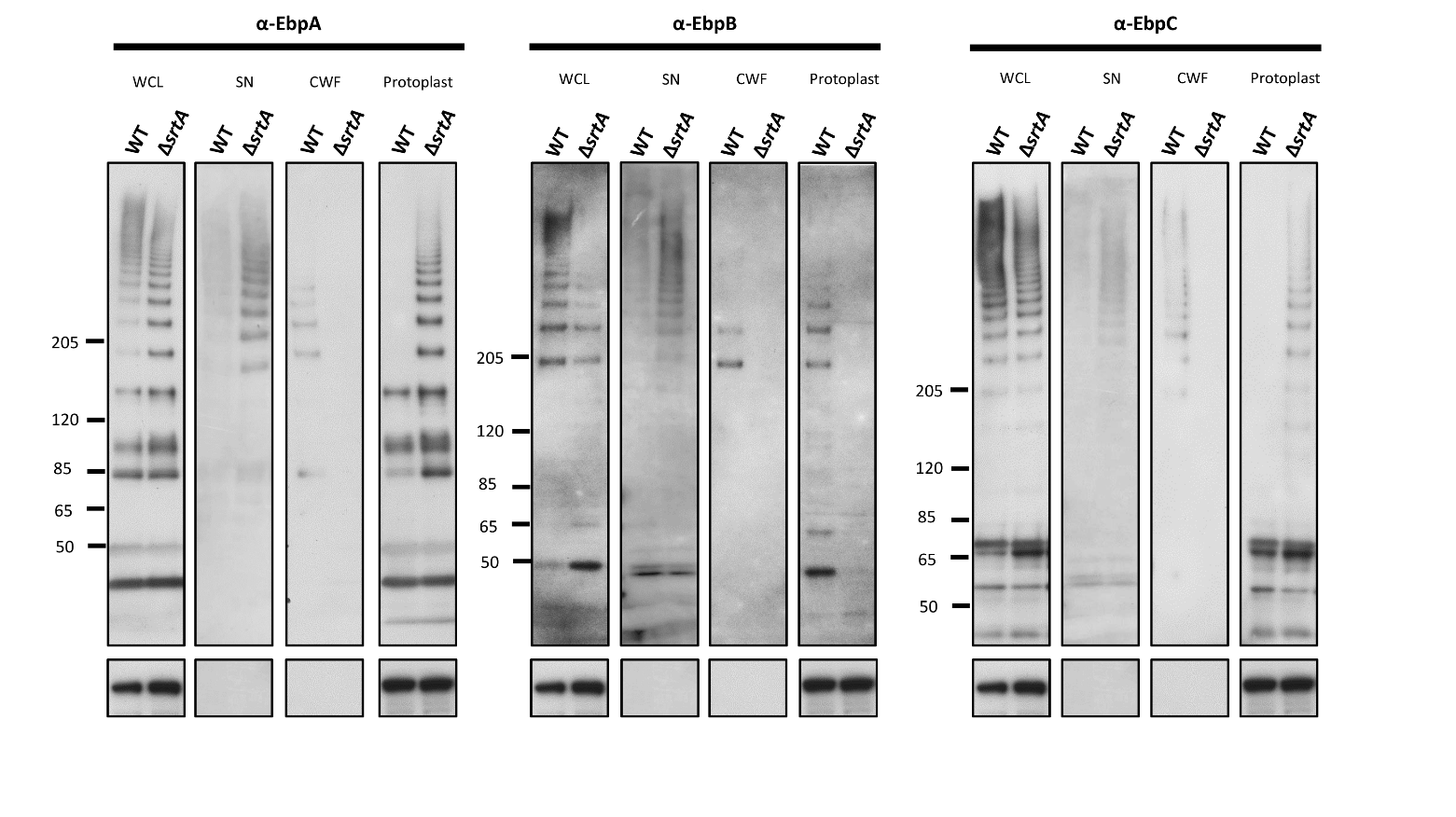


**Fig S2. Pili on WT cells are cell wall anchored while pili on Δ*srtA* are membrane anchored.** Ebp immunoblots of WT and Δ*srtA* cells in four fractions – whole cell lysate (WCL), supernatant fraction (SN), cell wall fraction (CWF) and protoplast fraction. All three Ebp subunits were blotted with SecA as a loading control. The predicted size of EbpA, EbpB, and EbpC monomers are 128 kDa ,53 kDa and 68 kDa respectively.


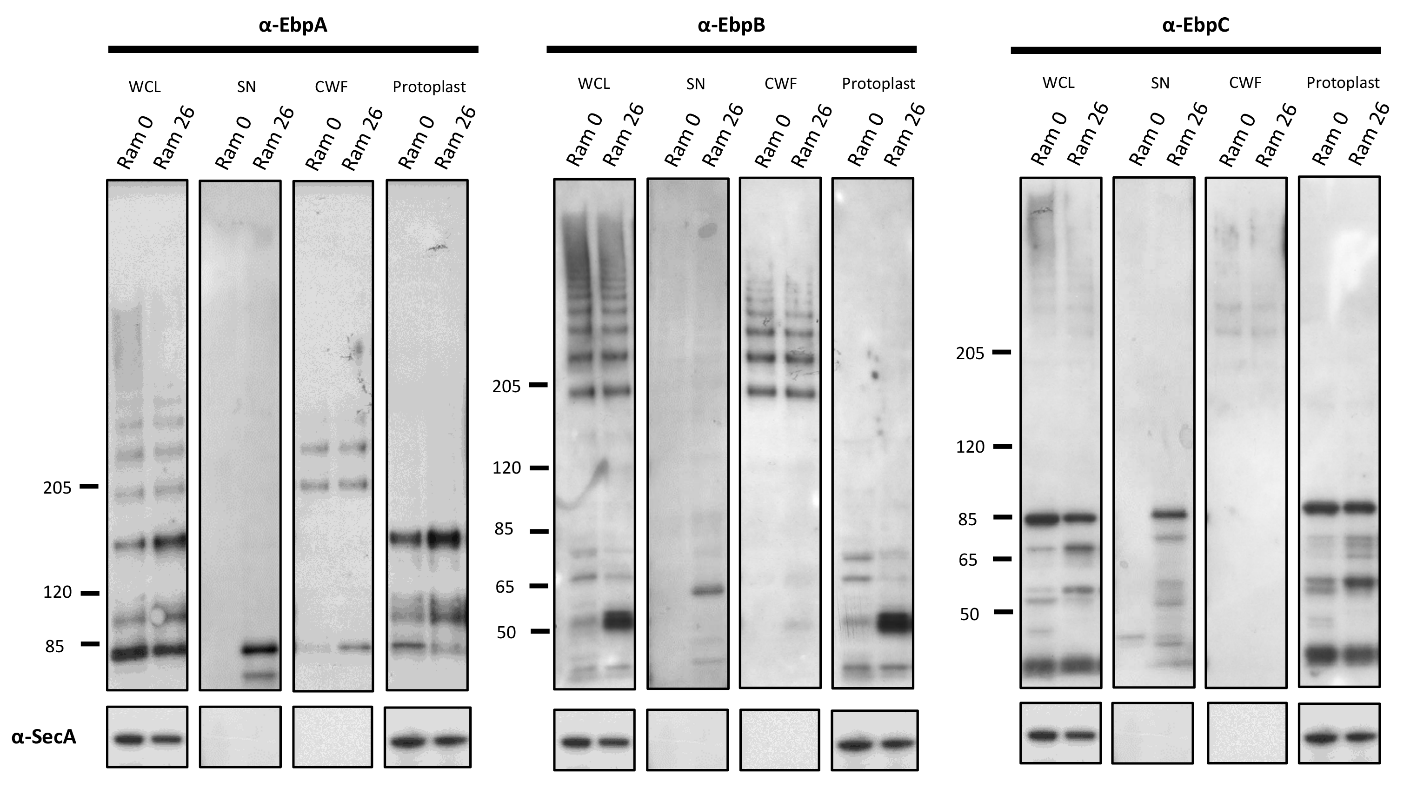


**Fig S3. New pili on ramoplanin treated cells are cell wall bound.** Ebp immunoblots of ramoplanin treated (Ram 26) and untreated (Ram 0) cells in four fractions – whole cell lysate (WCL), supernatant fraction (SN), cell wall fraction (CWF) and protoplast fraction. All three Ebp subunits were blotted with SecA as a loading control. The predicted size of EbpA, EbpB, and EbpC monomers are 128 kDa, 53 kDa and 68 kDa respectively.

**Table S1: Table of strains and plasmids**

| **Strain** | **Resistance** ^†^ | **Description** | **Reference or source** |
| --- | --- | --- | --- |
| OG1RF | Rif, Fus | Ebp+, SrtC+, SrtA+, AS− | (Dunny, Brown, & Clewell, 1978) |
| OG1RF*ΔsrtA* | Rif, Fus | Ebp+, SrtC+, SrtA−, AS− | (Guiton et al., 2009) |
| OG1RF*ΔsrtC* | Rif, Fus | Ebp+, SrtC−, SrtA+, AS− | (Nallapareddy et al., 2006) |
| OG1RFΔ*ebpABC* | Rif, Fus | Ebp−, SrtC+, SrtA+, AS− | (H. V. Nielsen et al., 2012) |
| OG1RF/pCF10 | Rif, Fus, Tet | Ebp+, SrtC+, SrtA+ , AS+ | (Afonina, Lim, Tan, & Kline, 2018) |
| **Plasmid** |  |  |  |
| pGCP123 | Kan | Parent plasmid | (H. V. Nielsen et al., 2012) |
| pGCP123 PsrtA SrtA-2L-mCherry | Kan | mCherry tagged SrtA with 2 amino acid linker | This study |
| pGCP123 PebpABC^K186A^ | Kan | Expression of EbpA, EbpB, and EbpC^K186A^ | (Hailyn V. Nielsen et al., 2013) |
| pAL1::SrtC-HA | Kan | HA tagged SrtC | (Kline et al., 2009) |
| ^†^Rif, rifampin; Fus, fusidic acid; Tet, tetracycline; Kan, kanamycin | | |  |

Afonina, I., Lim, X. N., Tan, R., & Kline, K. A. (2018). Planktonic Interference and Biofilm Alliance between Aggregation Substance and Endocarditis- and Biofilm-Associated Pili in *Enterococcus faecalis*. *J Bacteriol, 200*(24). doi:10.1128/JB.00361-18

Dunny, G. M., Brown, B. L., & Clewell, D. B. (1978). Induced cell aggregation and mating in Streptococcus faecalis: evidence for a bacterial sex pheromone. *Proc Natl Acad Sci U S A, 75*(7), 3479-3483. doi:10.1073/pnas.75.7.3479

Guiton, P. S., Hung, C. S., Kline, K. A., Roth, R., Kau, A. L., Hayes, E., . . . Hultgren, S. J. (2009). Contribution of autolysin and Sortase a during Enterococcus faecalis DNA-dependent biofilm development. *Infect Immun, 77*(9), 3626-3638. doi:10.1128/IAI.00219-09

Kline, K. A., Kau, A. L., Chen, S. L., Lim, A., Pinkner, J. S., Rosch, J., . . . Hultgren, S. J. (2009). Mechanism for sortase localization and the role of sortase localization in efficient pilus assembly in Enterococcus faecalis. *J Bacteriol, 191*(10), 3237-3247. doi:10.1128/JB.01837-08

Nallapareddy, S. R., Singh, K. V., Sillanpaa, J., Garsin, D. A., Hook, M., Erlandsen, S. L., & Murray, B. E. (2006). Endocarditis and biofilm-associated pili of *Enterococcus faecalis*. *Journal of Clinical Investigation, 116*(10), 2799-2807. doi:10.1172/jci29021

Nielsen, H. V., Flores-Mireles, A. L., Kau, A. L., Kline, K. A., Pinkner, J. S., Neiers, F., . . . Hultgren, S. J. (2013). Pilin and Sortase Residues Critical for Endocarditis- and Biofilm-Associated Pilus Biogenesis in Enterococcus faecalis. *Journal of Bacteriology, 195*(19), 4484-4495. doi:10.1128/jb.00451-13

Nielsen, H. V., Guiton, P. S., Kline, K. A., Port, G. C., Pinkner, J. S., Neiers, F., . . . Hultgren, S. J. (2012). The metal ion-dependent adhesion site motif of the Enterococcus faecalis EbpA pilin mediates pilus function in catheter-associated urinary tract infection. *MBio, 3*(4), e00177-00112. doi:10.1128/mBio.00177-12
